# Effective growth-suppressive activity of maternal embryonic leucine-zipper kinase (MELK) inhibitor against small cell lung cancer

**DOI:** 10.18632/oncotarget.7297

**Published:** 2016-02-10

**Authors:** Hiroyuki Inoue, Taigo Kato, Sope Olugbile, Kenji Tamura, Suyoun Chung, Takashi Miyamoto, Yo Matsuo, Ravi Salgia, Yusuke Nakamura, Jae-Hyun Park

**Affiliations:** ^1^ Department of Medicine, The University of Chicago, Chicago, IL 60637, USA; ^2^ OncoTherapy Science, Inc., Kawasaki, 213-0012, Japan

**Keywords:** small cell lung cancer, MELK, molecular target, kinase inhibitor, cancer stem cell

## Abstract

Maternal embryonic leucine zipper kinase (MELK), that plays a critical role in maintenance of cancer stem cells (CSCs), is predominantly expressed in various types of human cancer including small cell lung cancer (SCLC). SCLC usually acquires resistance to anti-cancer drugs and portends dismal prognosis. We have delineated roles of MELK in development/progression of SCLC and examined anti-tumor efficacy of OTS167, a highly potent MELK inhibitor, against SCLC. MELK expression was highly upregulated in both SCLC cell lines and primary tumors. siRNA-mediated MELK knockdown induced significant growth inhibition in SCLC cell lines. Concordantly, treatment with OTS167 exhibited strong cytotoxicity against eleven SCLC cell lines with IC_50_ of < 10 nM. As similar to siRNA knockdown, OTS167 treatment induced cytokinetic defects with intercellular bridges, and in some cell lines we observed formation of neuronal protrusions accompanied with increase of a neuronal differentiation marker (CD56), indicating that the compound induced differentiation of cancer cells to neuron-like cells. Furthermore, the MELK inhibition decreased its downstream FOXM1 activity and Akt expression in SCLC cells, and led to apoptotic cell death. OTS167 appeared to be more effective to CSCs as measured by the sphere formation assay, thus MELK inhibition might become a promising treatment modality for SCLC.

## INTRODUCTION

MELK (maternal embryonic leucine zipper kinase), also known as MPK38 (murine protein serine/threonine kinase 38), is involved in mammalian embryonic development and also functions as a cell-cycle dependent protein kinase in the cell mitosis phase [1, 2]. We previously reported MELK as a desirable therapeutic target to treat many types of cancer because of its indispensable roles in cancer cell survival and restricted expression in cancer cells [3]; *MELK* is highly expressed in a great majority of breast cancer and glioblastoma, but its expression was hardly detectable in normal adult tissues except in the testis [3, 4]. In addition, several studies have demonstrated that high expression of *MELK* was correlated with poorly differentiated phenotypes (malignancy grade) in human astrocytoma and prostate cancers, and is associated with poor prognosis of breast cancer patients [5].

It is also suggested that MELK is involved in the maintenance of cancer stem cells (CSCs), which possess higher tumorigenicity and are, in general, resistant to conventional anti-cancer therapies [6, 7]. Hence, therapeutic strategies to target the MELK in CSCs should overcome the drawbacks of the conventional anti-cancer therapies. Previously, we reported development of a potent MELK inhibitor (OTS167) that effectively abrogated MELK kinase activity and suppressed growth of human breast cancer cells and acute myeloid leukemia cells [8, 9]. Either intravenous injection or oral administration of OTS167 exhibited significant tumor growth suppressive effect on multiple human cancer xenograft models [9]. Our results also demonstrated that OTS167 significantly inhibited the formation of mammosphere derived from breast cancer cells [9], implicating that OTS167 could be very effective to suppress the growth of CSCs.

Small cell lung cancer (SCLC) comprises approximately 15% of all lung cancers that annually affects more than 200,000 people worldwide [10]. In general, SCLC exhibits aggressive behavior, rapid growth, and early spread to distant sites, which collectively contribute to high mortality rate [11]. Moreover, SCLC patients often have a metastasized lesion(s) at the time of diagnosis and their survival rate has been improved little over last three decades [12], indicating the importance of urgent development of novel effective treatment modalities. Etiologically, SCLC is thought to derive from self-renewing pulmonary neuroendocrine progenitors [13, 14]. It was reported that the MELK expression was elevated in neural progenitors and hematopoietic stem cells [15], and that overexpression of MELK enhanced the formation of neurospheres [16]. However, the involvement of MELK in SCLC has not yet been elucidated.

In current study, we demonstrate that MELK was overexpressed in the majority of SCLC cell lines and primary tumors, and that either knockdown of MELK or treatment with a MELK inhibitor (OTS167) exhibited growth inhibitory effect on all SCLC cell lines examined. Our results suggest that MELK is a promising therapeutic target for SCLC treatment and the MELK inhibitor OTS167 should be clinically assessed as a new class of anti-SCLC agents.

## RESULTS

### MELK is highly expressed in SCLC cell lines and primary SCLC tissues

**Figure 1:**
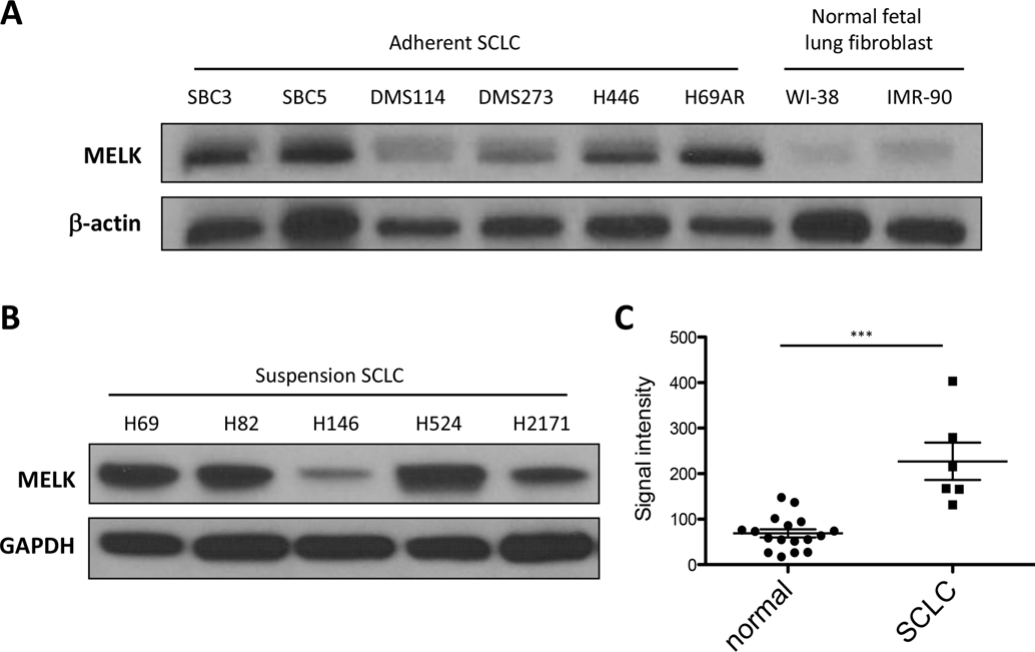
MELK is highly expressed in SCLC cell lines and primary SCLC tumors Endogenous MELK protein expression levels were examined by western blot analysis of 6 adherent SCLC cell lines, 2 NFLF (normal fetal lung fibroblast) cells (**A**), and 5 suspension SCLC cell lines (**B**). (**C**) The expression of *MELK* mRNA is significantly upregulated in primary SCLC tumors compared with that of normal lung tissues (***p < 0.001). Horizontal lines represent the means ± standard deviations.

To assess the MELK expression levels in SCLC, we performed immunoblot analyses using 11 human SCLC cell lines (six adherent cells and five suspension cells) and 2 normal fetal lung fibroblasts (NFLF) cell lines, and found that MELK protein was highly expressed in the majority of both adherent and suspension SCLC cell lines; whereas it was expressed in 2 NFLF normal counterparts at very low levels (Figure 1A and 1B). In addition, we performed comprehensive analysis of the *MELK* expression in various cancer cell lines using gene expression datasets from the Cancer Cell Line Encyclopedia (CCLE). The average expression level of *MELK* in 53 SCLC cell lines was high as being ranked to the 5th of 33 different cancer types (Supplementary Figure 1). Furthermore, the Oncomine database revealed that *MELK* expression in six primary SCLC tissues were significantly higher than that in 17 normal lung tissues (*p* < 0.001) [17] (Figure 1C).

### siRNA-mediated MELK knockdown decreases cell viability

**Figure 2:**
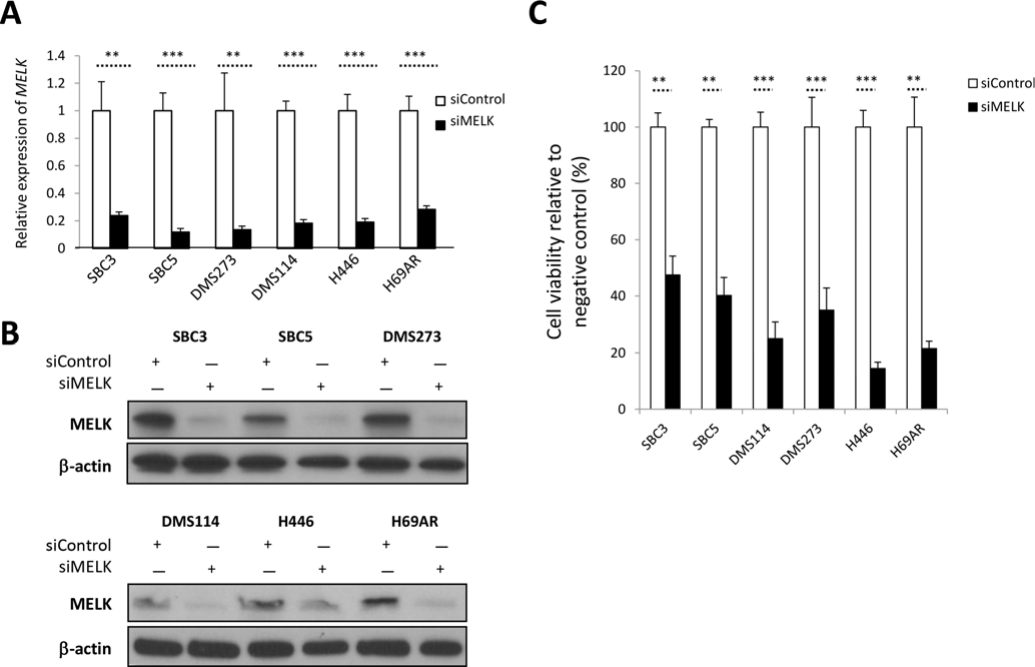
siRNA-mediated MELK knockdown results in decrease of cell viability in SCLC cells (**A**) Real-time RT-PCR analyses were conducted to examine expression levels of *MELK* in 6 adherent SCLC cells at 48 hours after transfection with control siRNA or MELK siRNA (**p < 0.01; ***p < 0.001). (**B**) Western blot analyses were performed to measure MELK protein levels in the cell lysates harvested from 6 adherent SCLC cells, 48 hours after transfection with control siRNA or MELK siRNA. (**C**) MTT assays were performed 6 days after transfection with siRNAs (**p < 0.01; ***p < 0.001).

Loss of function approach by siRNA-mediated knockdown of MELK confirmed significant decrease of *MELK* expression with MELK siRNA in six adherent SCLC cell lines, compared with those transfected with si-control by quantitative RT-PCR (**P < 0.01, ***P < 0.001) (Figure 2A). Immunoblot analyses showed the significant reduction of MELK protein in all six cell lines tested at 48 hours after si-MELK transfection (Figure 2B). MTT assay revealed significant decrease of the cell viability in these SCLC cell lines transfected with si-MELK, compared with those transfected with si-control (**p < 0.01 or ***p < 0.001) (Figure 2C). These findings suggested that MELK possibly plays critical roles in the proliferation and/or survival of SCLC cells.

### MELK inhibitor OTS167 displays anti-tumor activity in SCLC cell lines

**Figure 3:**
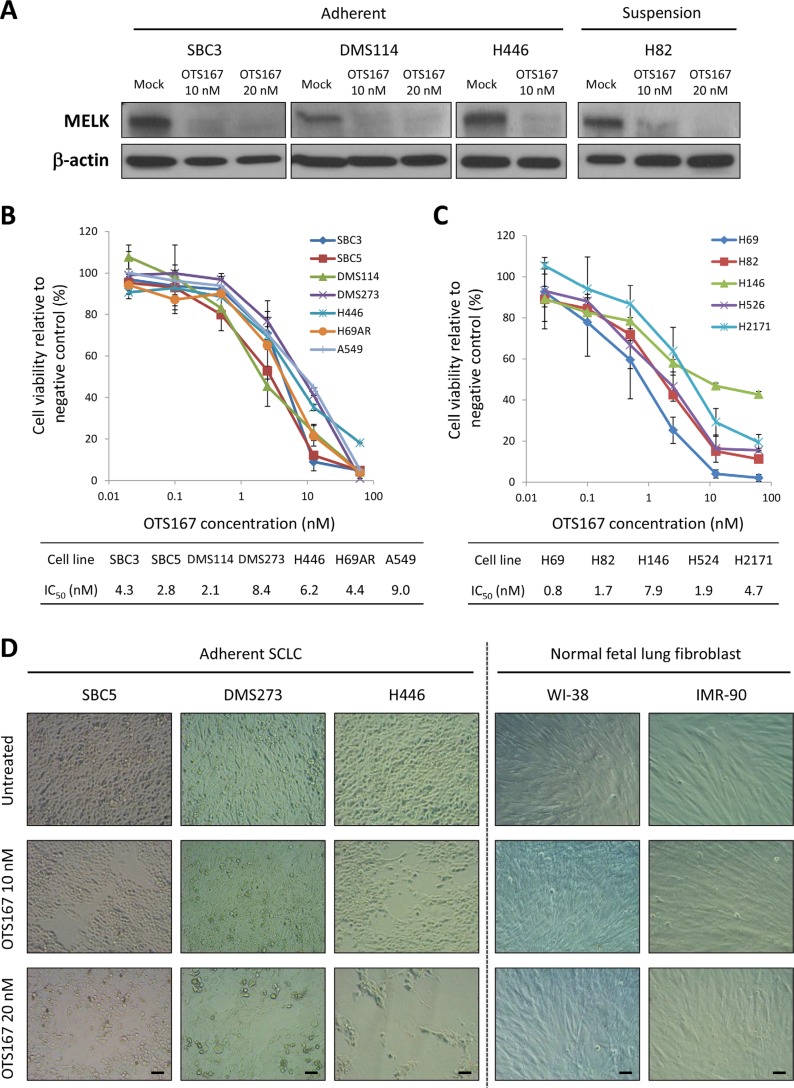
Treatment with MELK inhibitor shows strong growth-suppressive activity against SCLC cells but not normal NFLF cells (**A**) Western blot analyses were performed to measure MELK protein levels in adherent and suspension SCLC cells, 48 hours after treatment with OTS167 (0, 10, and 20 nM). MTT assay was performed in six adherent SCLC cells and one lung adenocarcinoma cell (**B**) or in five suspension SCLC cells (**C**). All cells were measured their viability after treatment with OTS167 at different concentrations (0 to 50 nM) for 72 hours. Graphs indicate relative cell viability at each OTS167 concentration, compared to a negative control (untreated). (**D**) The cytotoxic effects induced by OTS167 treatment (10 nM or 20 nM) in three adherent SCLC cells and two NFLF cells were evaluated by microscopic observation *(x 200 magnification)*. Scale bar indicates 50 μm.

As we have demonstrated that MELK knockdown resulted in the decrease in cell viability of SCLC cell lines, we then investigated growth-suppressive effects of a potent MELK inhibitor, OTS167 [9]. At first, we examined effects of OTS167 on MELK protein levels, because MELK was known to have autophosphorylation that contributes to the protein stability [18]. We treated four SCLC cell lines (SBC3, DMS114, H446, and H82) with 10 or 20 nM of OTS167 for 48 hours, and detected that OTS167 treatment reduced the MELK protein level (Figure 3A). OTS167 treatment exhibited strong growth-suppressive effects against all of the eleven SCLC cell lines with the half-maximum inhibitory concentration (IC_50_) values ranging from 0.8 nM to 8.4 nM (Figure 3B and 3C). Importantly, H446 cells harboring high CSC properties *in vitro* and *in vivo* [19], and H69AR cells, which are resistant to multi-cytotoxic agents [20], were also very sensitive to this compound with IC_50_ values of 6.2 nM and 4.4 nM, respectively. Microscopic observation clearly indicated that OTS167 treatment caused cytotoxic effects in SCLC cells in a dose-dependent manner, while 2 NFLF cell lines were not damaged at these concentrations (Figure 3D).

### MELK inhibitor manifests cytokinetic defects with increase of differentiation marker CD56

**Figure 4:**
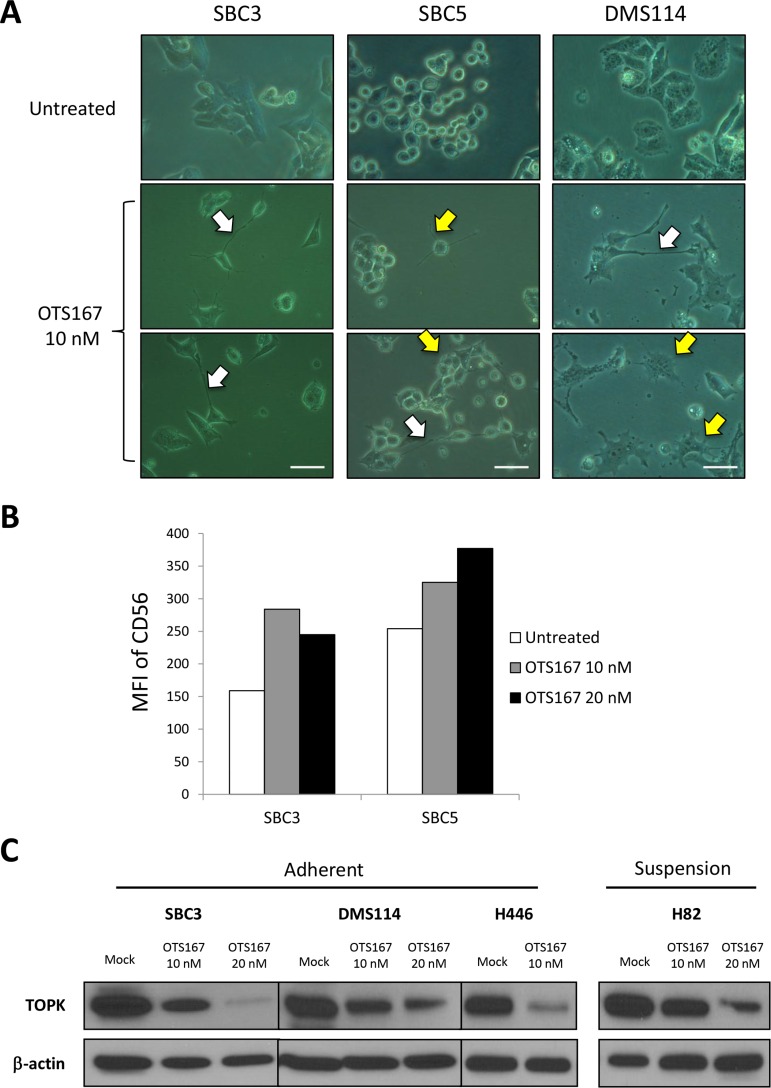
MELK inhibitor induces cytokinetic defects accompanied with differentiation into neuron-like cells (**A**) Three adherent SCLC cells were treated with 10 nM of OTS167, and microscopic observation was conducted 48 hours later *(x 400 magnification)*. A scale bar indicates 50 μm. Yellow arrows and white arrows depict neuronal protrusions and intercellular bridge formation, respectively. (**B**) Two adherent SCLC cells were treated with 10 nM and 20 nM of OTS167 and flow cytometry analysis was performed to detect CD56 protein expression levels after 48-hour treatment. Graph indicates the mean fluorescence intensity (MFI) corresponding to surface CD56 expression in SCLC cells. (**C**) Western blot analysis was performed to measure TOPK protein levels in untreated and OTS167-treated SCLC cells (10 nM and 20 nM).

We also examined morphological changes induced by a MELK inhibitor OTS167 in SCLC cells and observed that MELK inhibitor-treated SCLC cells revealed dendrite-like neuronal protrusions (yellow arrows) as well as elongated intercellular bridges (white arrows), which were not observed in SCLC cells without the exposure to OTS167 (Figure 4A). Similar morphological changes including the cytokinetic defects were observed by knockdown of MELK, supporting that they were caused by suppression of MELK activity (Supplementary Figure 2). Since SCLC is considered to derive from self-renewing pulmonary neuroendocrine progenitor cells [13, 14], we hypothesized that MELK inhibition might facilitate neuronal differentiation in SCLC cell lines. Therefore, we examined expression of a neural differentiation maker, NCAM (neural cell adhesion molecule, also called CD56) using flow cytometry in SCLC cells treated with OTS167. Expectedly, the MELK inhibitor treatment increased expression of CD56 in SCLC cells (Figure 4B) as well as proportions of CD56-positive cells (Supplementary Figure 3). We previously reported the cytokinetic defects accompanied with intercellular bridges by inhibition of T-LAK cell-originated protein kinase (TOPK) [21]. It was also shown that both MELK and TOPK are putative CSC markers as included in top 20 of the “consensus stemness ranking (CSR) signature” genes [22]. In light of these findings, we examined relationship between MELK and TOPK by quantifying TOPK protein level in SCLC cells treated with OTS167. Our results revealed that treatment with OTS167 decreased the TOPK protein level in SCLC cells (Figure 4C) as well as the transcriptional level of *TOPK* (Supplementary Figure 4) (*p < 0.05, **p < 0.01). Concordantly, siRNA-mediated knockdown of *MELK* decreased TOPK both at protein level and at transcriptional level (*p < 0.05, **p < 0.01) (Supplementary Figure 5). Furthermore, in the analyzed data set of cancer patients, it was found that *MELK* expression was strongly correlated with that of *TOPK* in various types of cancers such as prostate adenocarcinoma and renal clear cell carcinoma (Pearson's correlation coefficient *R* values are 0.82 and 0.81, respectively), and moderately correlated with lung adenocarcinoma (*R* = 0.55) (Supplementary Figure 6).

### MELK inhibitor downregulates FOXM1 activity and decreases Akt protein

**Figure 5:**
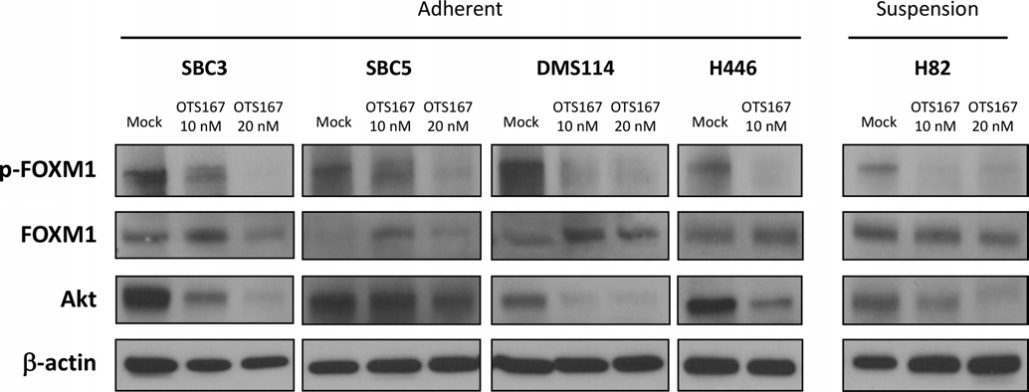
Treatment with MELK inhibitor downregulates FOXM1 activity and Akt expression in SCLC cells Western blot analyses were performed to measure protein levels of total FOXM1, phosphorylated FOXM1, and Akt in both adherent and suspension SCLC cells treated with 10 nM or 20 nM of OTS167 for 48 hours.

To further understand the mechanism of action of OTS167, we examined possible downstream molecules of MELK in SCLC cells. At first, we investigated FOXM1 activity because FOXM1 was identified as an important substrate of MELK in glioma stem cells [23] and its activity was attenuated by OTS167 in acute myeloid leukemia cells [8]. We found that the active form of FOXM1, phosphorylated FOXM1 protein, was reduced in SCLC cells treated with OTS167 in a dose-dependent manner (Figure 5). We also examined an Akt-signaling pathway in SCLC cells since the PI3K/AKT/mTOR pathway is frequently activated in SCLC tumors [24] and exogenous introduction of FOXM1 activates the AKT pathway in breast cancer cells [25]. Our immunoblot results clearly demonstrated that treatment with OTS167 decreased the total Akt protein level in both adherent and suspension SCLC cells (Figure 5).

### Caspase-mediated apoptosis is attributed to OTS167-mediated growth suppressive effects in SCLC cells

**Figure 6:**
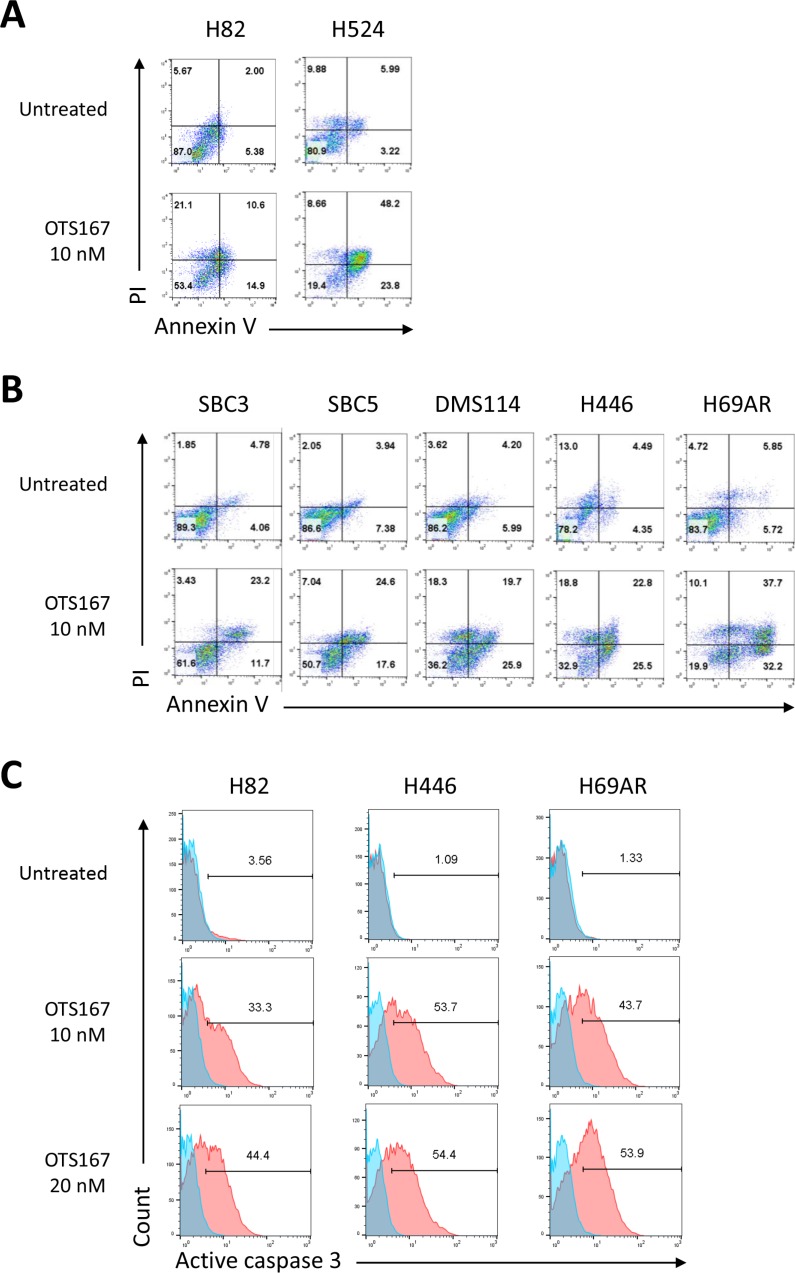
Treatment with MELK inhibitor results in substantial apoptosis in SCLC cells Two suspension (**A**) and five adherent (**B**) SCLC cell lines were treated with 10 nM of OTS167. At 48 hours of the treatment, Annexin-V and PI staining assay was performed to detect an early phase of apoptosis. The numbers depict the percentage of cells in each quarter. (**C**) At 60 hours of treatment with 10 or 20 nM of OTS167, flow cytometry analysis was conducted to comparatively quantify levels of cleaved caspase 3 for assessment of a late phase of apoptosis in SCLC cells. The numbers depict the percentage of cells in each gate.

To address the mechanism of the growth suppressive effects by OTS167, we further evaluated its effects on cancer cell death and found by flow cytometry analyses that treatment with OTS167 exhibited a greater proportion of early apoptotic cells (Annexin-V+/PI-) as well as necrotic (Annexin-V+/PI+) cells in both suspension and adherent SCLC cells (Figure 6A and 6B). We then explored the use of the active (cleaved) form of caspase-3 for the detection of the execution phase of apoptotic events. OTS167 treatment markedly induced activation of caspase-3 in a dose-dependent manner, in both suspension and adherent SCLC cells (Figure 6C). Moreover, apoptosis induced in SCLC cells by OTS167 was significantly abrogated by pre-treatment with a pan-caspase inhibitor (z-VAD-FMK) in a dose-dependent manner (Supplementary Figure 7), suggesting that OTS167 treatment induced the caspase-dependent apoptosis in SCLC cells.

### OTS167 treatment preferentially suppresses the lung sphere formation

**Figure 7:**
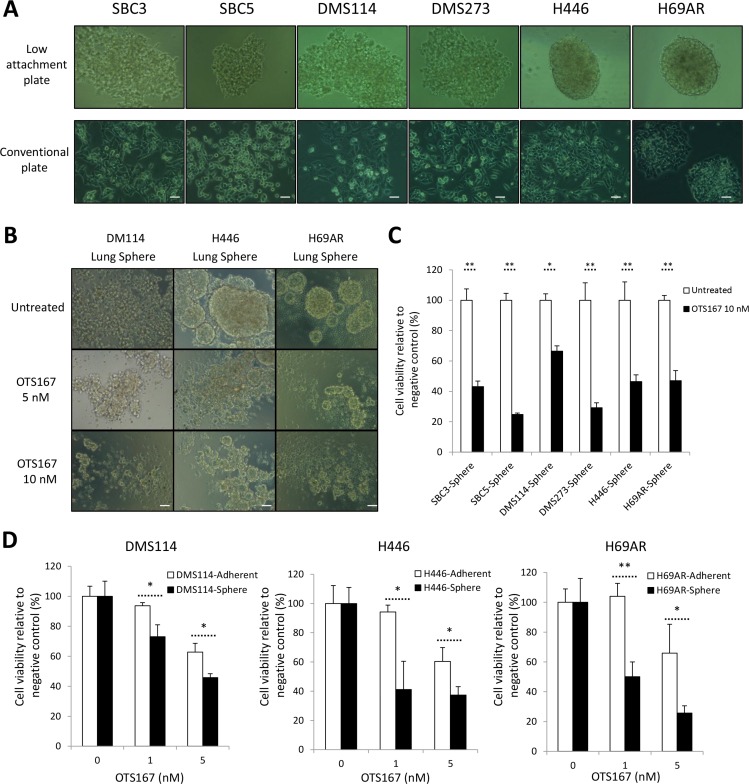
Treatment with MELK inhibitor effectively suppresses formation of lung sphere (LS) (**A**) Six adherent SCLC cells were seeded onto conventional culture plate or ultra-low attachment culture plate. After 15 days of culture, LS formation in ultra-low attachment culture plate was measured by microscopic observation (*x 200 magnification*). (**B**) After dissociation of LS into single cells by mechanical pipetting, these LS-derived SCLC cells were cultured with or without 5 nM or 10 nM of OTS167. After 48 hours of treatment, microscopic observation was conducted to assess morphological changes caused by OTS167 (*x 200 magnification*). A scale bar indicates 50 μm. (**C**) At the same time point, MTT assays were performed to evaluate the growth-suppressive effect of OTS167 (10 nM) on LS formation derived from 6 adherent SCLC cells. Graphs indicate relative cell viability of each LC, compared to untreated control cells (*p < 0.05, **p < 0.01). (**D**) MTT assays were performed to compare the growth-suppressive effect of OTS167 treatment (5 nM or 10 nM) between adherent SCLC cells and LS-derived SCLC cells cultured in normal culture plates for 48 hours. Graphs indicate relative cell viability at each OTS167 concentration, compared to untreated control cells (*p < 0.05, **p < 0.01).

SCLC cells can grow as spheres that are enriched with cancer stem-like cells harboring increased *in vitro* clonogenic and *in vivo* tumorigenic potentials [26]. Hence, we conducted lung sphere (LS) formation assay using six adherent SCLC cell lines. LS formation was developed through serial passage of cancer cells under low attachment culture condition (no LS formation was observed under a method using conventional culture plates) (Figure 7A). To characterize the selected SCLC cells by the LS formation, we examined expression of CD133, one of the CSC markers in SCLC cells [27]. We found that LS-derived SCLC cells displayed elevated expression of CD133, compared with parental SCLC cells (Supplementary Figure 8). Other reports implicated that MELK could play essential roles in maintenance of CSCs for breast cancer and glioblastoma cells, and therefore be an attractive target to eradicate CSCs [6, 28]. As expected, treatment with OTS167 decreased the proportion of CD133-expressing cancer cells in both adherent and suspension SCLC cell lines (Supplementary Figure 9). Furthermore, we demonstrated that, in LS formation assay using six LS-derived SCLC cells, treatment with OTS167 significantly suppressed the development of LS formation in all SCLC cells examined (Figure 7B and 7C). Subsequently, we compared the sensitivity to OTS167 treatment between the LS-derived SCLC cells and parental adherent SCLC cells by MTT assays, and identified that OTS167 treatment more significantly suppressed the growth of LS-derived SCLC cells than that of parental adherent SCLC cells (Figure 7D), further suggesting a possibility that OTS167 treatment may effectively work on CSCs.

## DISCUSSION

It has been reported that MELK is frequently and highly upregulated in various types of human cancer, both solid tumors and hematological cancers, and that elevated MELK expression is correlated with poor prognosis of cancer patients [5]. However, involvement of MELK in carcinogenic process of SCLC has never been scrutinized. In this study, we demonstrated that most of SCLC cell lines as well as primary SCLC tumors highly expressed MELK. In addition, public datasets revealed very high level of *MELK* expression in SCLC among 33 different cancer types examined. These results implied that the MELK might play essential roles in development/progression of SCLC and be an excellent target for development of anti-SCLC drugs.

We investigated the role of MELK in the growth of SCLC cells through two loss-of-function approaches, one targeting *MELK* expression with siRNA and the other targeting the MELK kinase activity with a small molecule inhibitor OTS167. Both approaches clearly indicated that MELK protein is critically important for the proliferation and/or survival of SCLC cells. Since treatment with OTS167 hardly displayed cytotoxic effects on 2 NFLF cells, we expect that this compound could selectively eradicate tumors with no or minimum adverse effect on normal cells. Indeed, we previously reported that treatment with the MELK inhibitor (OTS167) effectively suppressed growth of tumors in xenograft mice models, but did not reveal any detectable adverse reactions at the effective dose [9].

Our immunoblot analyses showed that treatment with the MELK inhibitor significantly inhibited the FOXM1 activity and decreased the pan-Akt protein level. FOXM1 is known to be involved in angiogenesis, invasion, metastasis, and drug resistance [29–31]. It is also reported that FOXM1 can activate the PI3K/Akt/mTOR pathway which is one of dominant oncogenic pathways in human cancer [25]. The PI3K/Akt/mTOR pathway is also frequently activated in SCLC tumors by loss-of-function mutations in *PTEN* or by activating alterations in *PI3K* genes [24]. Therefore, treatment with MELK inhibitor could be a desirable anticancer strategy for tumors with enhanced activity in the PI3K/Akt/mTOR pathway, such as breast, lung, ovarian, and prostate cancers [32].

Intriguingly, treatment with OTS167 induced cytokinetic defects accompanied by reduction of TOPK protein level in SCLC cells. This phenomenon was further confirmed by siRNA-mediated MELK knockdown, which could exclude a possibility of off-targeting effects by this compound. In addition, co-expression of *MELK* and *TOPK* genes in different types of cancers suggests an interaction between *MELK* and *TOPK*. Indeed, our findings demonstrated that inhibition of MELK downregulated transcriptional expression of *TOPK* in SCLC cells. It might be caused by FOXM1 which plays oncogenic roles as a transcriptional factor and can bind to a promoter region of the *TOPK* gene [33]. Since FOXM1 was inactivated by OTS167 treatment, our results implied that the MELK inhibitor might diminish FOXM1-mediated transcriptional induction of the *TOPK* gene in SCLC cells.

The capacity of OTS167 to induce proapoptotic changes, even in the CSC-like H446 cells, is very attractive for the treatments of refractory SCLC because suppression of apoptosis is considered as one of the hallmarks of cancer cells and CSC generally have higher levels of the anti-apoptotic proteins [34]. Our results of OTS167-mediated cytotoxicity in human SCLC cells were dependent on the caspase cascade, implying that OTS167 treatment could lead to other multimodal programmed cancer cell deaths such as necroptosis, pyroptosis and autophagic cell death [35], although further assessments are required.

Since MELK was suggested to be one of key molecules that maintain characteristics of CSC, we explored the anti-tumor effect of OTS167 on the formation of LS as a preclinical model that recapitulates lung CSC. OTS167 treatment significantly inhibited the LS formation in a dose-dependent manner. More importantly, the comparison of MTT assays in parental adherent SCLC cells and LS-derived SCLC cells revealed that OTS167 more effectively suppressed the growth of the latter cells as MELK inhibition by siRNA or OTS167 on breast cancer cells reported previously [9]. We also observed that treatment with OTS167 enhanced expression of CD56, a marker of neuronal cell differentiation. Thus, MELK-targeting therapy could be applied as a differentiation therapy, which aims to force the SCLC cells to resume the neuronal differentiation from progenitor phenotype.

In conclusion, we have elucidated that MELK plays pivotal roles in cancer progression and/or stem cell maintenance in SCLC cells. Our data have also demonstrated that the selective MELK inhibitor OTS167 could attenuate the FOXM1 and Akt pathways leading to caspase-dependent apoptosis of SCLC cells, and possesses strong *in vitro* growth suppressive effects on CSC-mimic SCLC cell subpopulations. Our results indicate that the MELK inhibitor OTS167 has a great potential for the treatment of SCLC patients who did not respond to conventional therapies.

## MATERIALS AND METHODS

### Cell lines

SBC-3 and SBC-5 cells were purchased from Japanese Collection of Research Bioresources Cell Bank (JCRB) (Suita, Japan). Adherent SCLC (DMS114, H69AR and H446) cell lines, suspension SCLC (H69, H82, H146, H524 and H2171) cell lines, A549 lung adenocarcinoma and normal lung fetal lung fibroblasts (IMR-90 and WI-38) cell lines were purchased from the American Type Culture Collection (ATCC) (Manassas, VA). DMS273 cell line was purchased from European Collection of Authenticated Cell Cultures (ECACC, Salisbury, UK). SBC3, SBC5, IMR-90 and WI-38 cell lines were cultured in EMEM medium supplemented with 10% fetal bovine serum (FBS) (Life Technologies, Grand Island, NY) and 1% antibiotic-antimycotic solution (Sigma-Aldrich, St. Louis, MO). Other SCLC cell lines and A549 cells were cultured in RPMI medium supplemented with 10% FBS. All cells were maintained at 37°C in humidified air with 5% CO_2_. Cell authentication result for majorly used SCLC cell lines (SBC3, SBC5, DMS114, H446, H82 and H524) was described in Supplementary Table 1.

### Transfection of siRNAs

For knockdown experiments, SCLC cell lines were transiently transfected with 200 pmol of oligo siRNA using Lipofectamine RNAiMAX (Invitrogen, Carlsbad, CA) according to manufacturer's instructions. The siRNA targeting (si-MELK, 5′-GACAUCCUAUCUAGCUGCA-3′) and a universal negative control (si-control) were purchased from Sigma-Aldrich.

### Western blot analysis and antibodies

Cells were lysed with IP lysis buffer (Thermo Scientific, Waltham, MA) containing protease inhibitor cocktail III (EMD Millipore, Billerica MA). The proteins were separated by electrophoresis using 4–20% SDS-PAGE gel (Bio-Rad, Hercules, CA) and transferred onto PVDF membranes. The membranes were incubated with the first antibody, respectively: anti-TOPK antibody (BD Biosciences, Franklin Lakes, NJ), anti-FOXM1 antibody (Santa Cruz Biotechnology, Dallas, TX), anti-pan-Akt (Cell Signaling, Danvers, MA), anti-β-actin (Sigma-Aldrich), or anti-GAPDH antibody (Sigma-Aldrich). A mouse anti-MELK monoclonal antibody against partial MELK protein (264–601 amino acids) was used to detect MELK protein, as described previously [9]. β-actin or GAPDH was used as a loading control.

### Quantitative RT-PCR

Total RNA was extracted from cancer cells using RNeasy Mini Kit (Qiagen, Valencia, CA) according to the manufacturer's directions. Total RNA (1–2 μg each) was reversely transcribed using SuperScript III First-Strand Synthesis System (Invitrogen) following the manufacturer's instructions. Aliquots of the reversely-transcribed product were quantified by real-time RT-PCR. The RT-PCR was performed using primers listed below using the ViiA 7 system (Life Technologies, Grand Island, NY). The expression levels were normalized with that of *GAPDH*. The PCR primer sequences are 5′-AGACCCTAAAGATCGTCCTTCTG-3′ and 5′-GTG TTTTAAGTCAGCATGAGCAG-3′ for *TOPK*; 5′-GCTGC AAGGTATAATTGATGGA-3′ and 5′-CAGTAACATAAT GACAGATGGGC-3′ for *MELK*; and 5′-CGACCACTTTG TCAAGCTCA-3′ and 5′-GGTTGAGCACAGGGTACTT TATT-3′ for *GAPDH*.

### Cell viability analyses

For methyl thiazolyl tetrazolium (MTT) assay to assess cell viability, 4 × 10^3^ of adherent SCLC cells or 4 × 10^4^ of suspension SCLC cells were seeded into 96-well flat plates. Cells treated with OTS167 or those transfected with oligo siRNAs were cultured for 72 hours at 37°C under 5% CO_2_. OTS167 was provided by OncoTherapy Science, Inc (Kawasaki, Japan) [9]. When using LS-derived cells, cells treated with OTS167 were cultured for 48 hours. A pan-caspase inhibitor, z-VAD-FMK was purchased from Selleck Chemicals (Houston, TX). Cell counting kit-8 (Dojindo Molecular Technologies, Inc., Kumamoto, Japan) was used for MTT reaction. To quantify cell viability, the 96-well plate was read at 450 nm of wavelength in the iMark microplate absorbance reader (Bio-Rad) after reaction for an hour. All of these experiments were done in triplicate.

### Flow cytometry analyses

For Annexin-V/PI staining analysis, SCLC cells treated with DMSO or OTS167 were collected, spun down, washed with PBS, and then resuspended in 50 μL of binding buffer containing 2 μL of APC-conjugated Annexin-V antibody (eBioscience, San Diego, CA). After 20 min incubation, the cells were stained with 100 μL of binding buffer containing 1 μL of propidium iodide (PI) (eBioscience). For detection of cleaved caspase 3, the cells were collected, spun down, washed with PBS, resuspended in 500 μL of Cytofix/Cytoperm solution (eBioscience), and then incubated for 20 min on ice. After washing with Perm/Wash buffer (eBioscience), the cells were resuspended with 100 μL of buffer containing 20 μL of PE-conjugated anti-cleaved caspase 3 antibody (eBioscience). For neuronal differentiation detection, SCLC cells treated with DMSO or OTS167 were washed with PBS, spun down, and stained with anti-human CD56 antibody (eBioscience) for 20 min at room temperature. Samples were subjected to flow cytometry instruments (FACS Calibur or FACS LSRII; Becton Dickinson, San Jose, CA) and analyzed using Flow Jo software (Treestar, Ashland, OR). To measure expression levels of CD133 surface protein, LS-derived SCLC cells and parental adherent SCLC cells were cultured in normal plate and stained with PE-conjugated anti-human CD133 antibody (AC133) (Miltenyi Biotec, San Diego, CA) for 15 min at room temperature. After washing with PBS, the cells were subjected to the flow cytometry instrument.

### Sphere formation assays

For examination of lung sphere formation under treatment with MELK inhibitor OTS167, 1 × 10^4^ of adherent SCLC cells were seeded onto the ultra-low attachment 96-well plate (Corning, Lowell, MA) and cultured for 8 days at 37°C under 5% CO_2_. Subsequently, through gentle pipetting, the detached SCLC cells were transferred into another ultra-low attachment 96-well plate for additional 7-day culture. Then LS formation was examined by an inverted microscope Axio Vert.A1 TL (Carl Zeiss Microscopy, Thornwood, NY).

### Gene expression dataset analysis

Expression levels of *MELK* and *TOPK* gene were investigated by use of publically available expression datasets that were deposited in the cBio Cancer Genomics Portal [36].

### Statistical analyses

Data were expressed as mean ± one standard deviation. Differences between two groups were examined for significance using student's *t*-test. Differences at *p* value of < 0.05 were considered to be significant.

## SUPPLEMENTARY MATERIALS FIGURES AND TABLE


