# Supplementary material for: Effective growth-suppressive activity of maternal embryonic leucine-zipper kinase (MELK) inhibitor against small cell lung cancer
Source: Oncotarget. 2016 Feb 10;7(12):13621–33. doi: 10.18632/oncotarget.7297 (PMC4924666; doi:10.18632/oncotarget.7297)
Supplement: Supplementary file 1 [file oncotarget-07-13621-s001.pdf]

Effective growth-suppressive activity of maternal embryonic leucine-zipper kinase (MELK) inhibitor against small cell lung cancer

Supplementary Materials

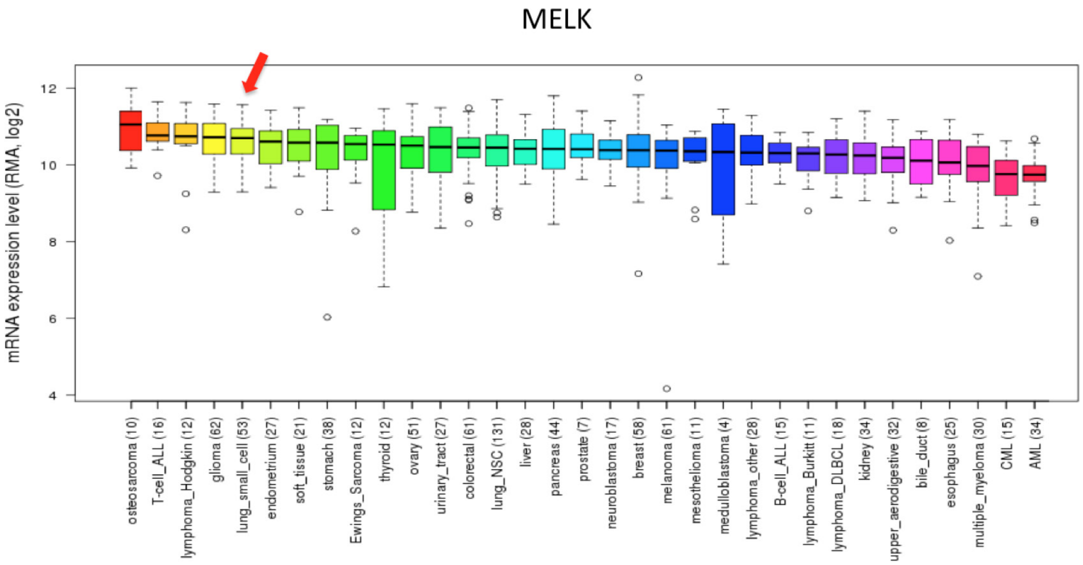

Supplementary Figure S1: MELK is highly up-regulated in SCLC cell lines. Gene-centric RMA-normalized mRNA expression in cancer cells obtained from Cancer Cell Line Encyclopedia (www.broadinstitute.org/ccle/home). SCLC cell lines (*red arrow*) significantly highly expressed *MELK*, compared to other cancer cell lines.

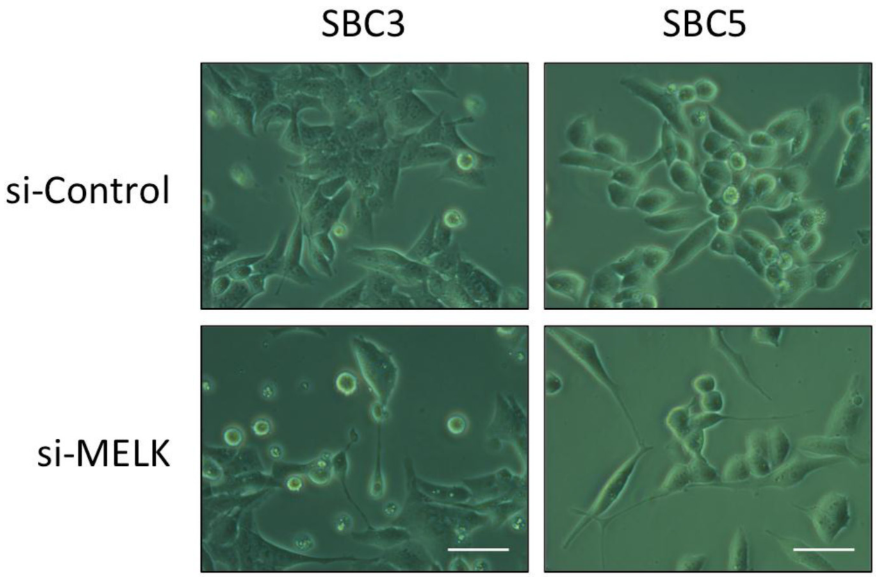

Supplementary Figure S2: siRNA-mediated MELK knockdown induces cytokinetic defect in SCLC cells. SBC3 and SBC5 cells transfected with si-control or si-MELK were cultured for 48 hours, and microscopic observation was conducted to examine morphological changes. A scale bar indicates 50 µm.

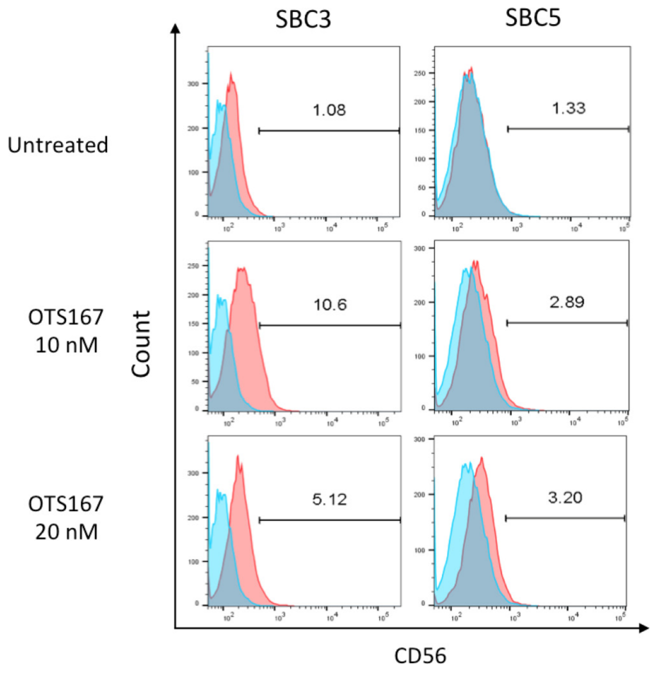

Supplementary Figure S3: Treatment with MELK inhibitor increases frequency of neuronal differentiation marker in SCLC cells. SBC3 and SBC5 cells were treated with 10 nM or 20 nM of OTS167, and then flow cytometry analysis was performed to measure the proportion and levels of CD56 protein expression, after 48 hours of OTS167 treatment.

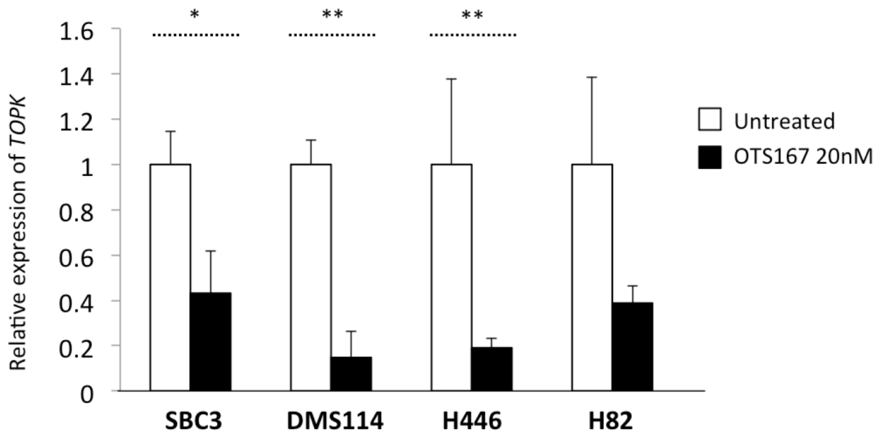

Supplementary Figure S4: Treatment with MELK inhibitor downregulates TOPK expression level in SCLC cells. Four SCLC cell lines were treated with OTS167, and then real-time RT-PCR analyses were performed to quantify the transcriptional level of *TOPK* after 48 hours of OTS167 treatment (*$p < 0.05$, **$p < 0.01$).

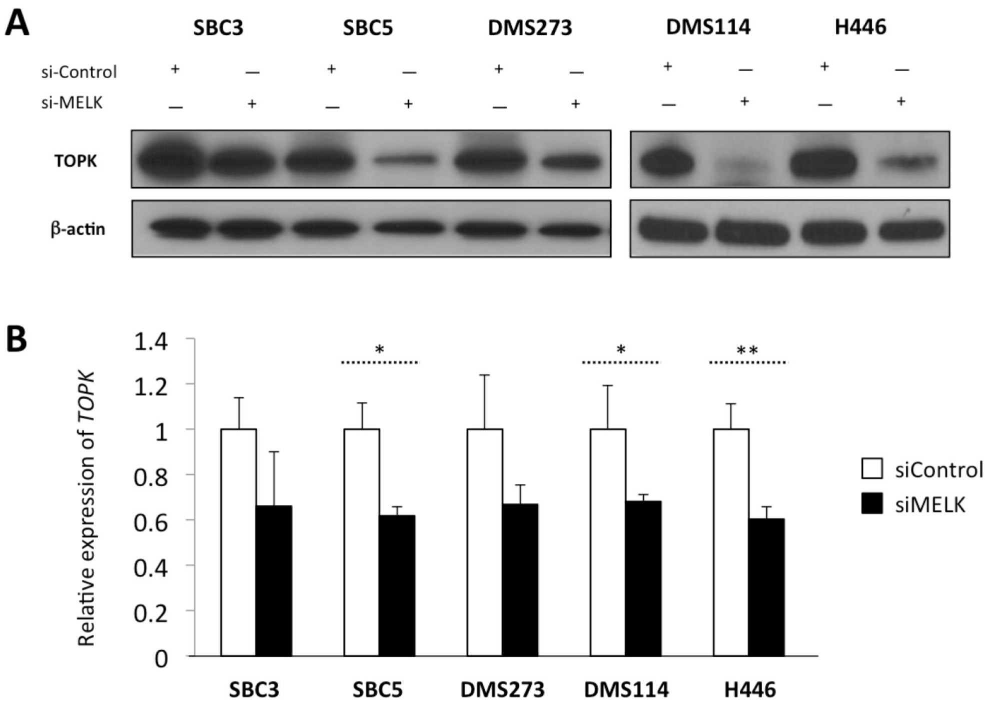

Supplementary Figure S5: siRNA-mediated MELK knockdown decreases TOPK protein level in SCLC cells. (**A**) Western blot analyses were performed to measure TOPK protein levels in the cell lysates harvested from 5 SCLC cells, at 48 hours after transfection with si-control or si-MELK. (**B**) Real-time RT-PCR were performed to assess the transcriptional level of *TOPK* in 5 SCLC cells at 48 hours after transfection with si-control or si-MELK (*$p < 0.05$, **$p < 0.01$).

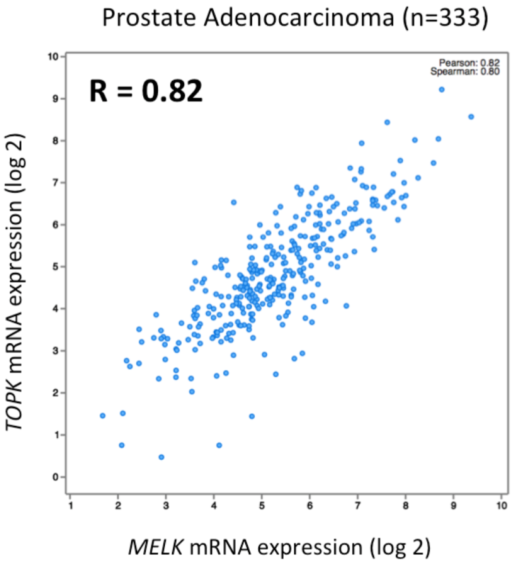

Type of Cancer	Pearson Correlation (R score)	N	Dataset
Prostate Adenocarcinoma	0.82	333	Cell. 2015 in press
Kidney Renal Clear Cell Carcinoma	0.81	392	Nature. 2013;499(7456):43-9.
Glioblastoma	0.80	91	Nature. 2008;455(7216):1061-8.
Sarcoma	0.80	149	Nat Genet. 2010;42(8):715-21.
Papillary Thyroid Carcinoma	0.74	388	Cell. 2014;159(3):676-90.
Liver Hepatocellular Carcinoma	0.73	190	deposited at NCI DB
Uterine Corpus Endometrioid Carcinoma	0.72	232	Nature. 2013;497(7447):67-73.
Breast Invasive Carcinoma	0.70	463	Nature. 2012;490(7418):61-70.
Stomach Adenocarcinoma	0.68	258	Nature. 2014;513(7517):202-9.
Pancreatic Adenocarcinoma	0.64	145	deposited at NCI DB
Ovarian Serous Cystadenocarcinoma	0.63	316	Nature. 2011;474(7353):609-15.
Lung Adenocarcinoma	0.55	230	Nature. 2014;511(7511):543-50.
Colorectal Adenocarcinoma	0.54	195	Nature. 2012;487(7407):330-7.
Cancer Cell Line Encyclopedia	0.50	877	Nature. 2012;483(7391):603-7.
Acute Myeloid Leukemia	0.47	163	deposited at NCI DB
Skin Cutaneous Melanoma	0.41	278	deposited at NCI DB
Bladder Urothelial Carcinoma	0.40	126	deposited at NCI DB
Head and Neck Squamous Cell Carcinoma	0.37	279	deposited at NCI DB

Supplementary Figure S6: Co-expression of *MELK* and *TOPK* genes in human cancers. Publically available gene expression datasets were analyzed to investigate co-expression of *MELK* and *TOPK* genes in various types of human cancers. A representative dataset of prostate adenocarcinoma (n = 333) showed significant correlation in the expression levels of both genes (Pearson Correlation, R = 0.82).

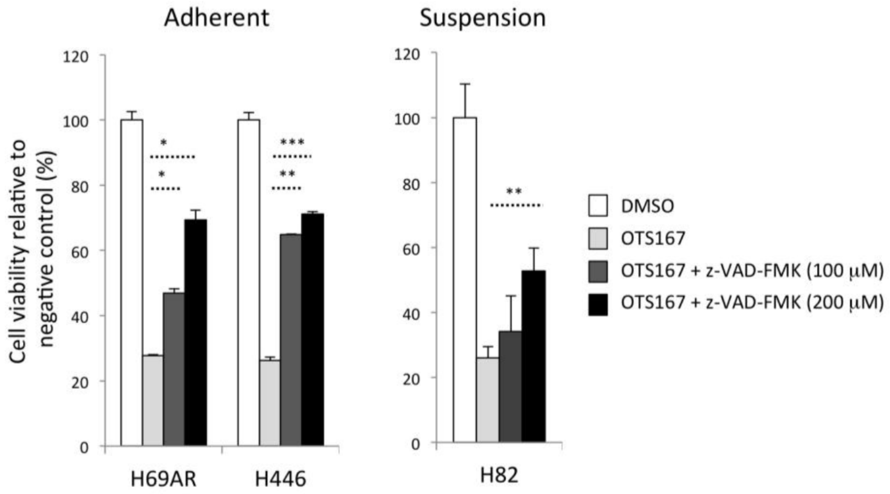

Supplementary Figure S7: Restoration of OTS167-induced apoptosis in SCLC cells by pan-caspase inhibitor. Adherent (H69AR and H446) and suspension (H82) SCLC cells were pre-treated with a pan-caspase inhibitor (z-VAD-FMK) for an hour, before treatment with 25 nM of OTS167 or DMSO for additional 72 hours. Relative cell viability to a negative control (DMSO) was assessed by MTT assay (*$p < 0.05$, **$p < 0.01$, ***$p < 0.001$).

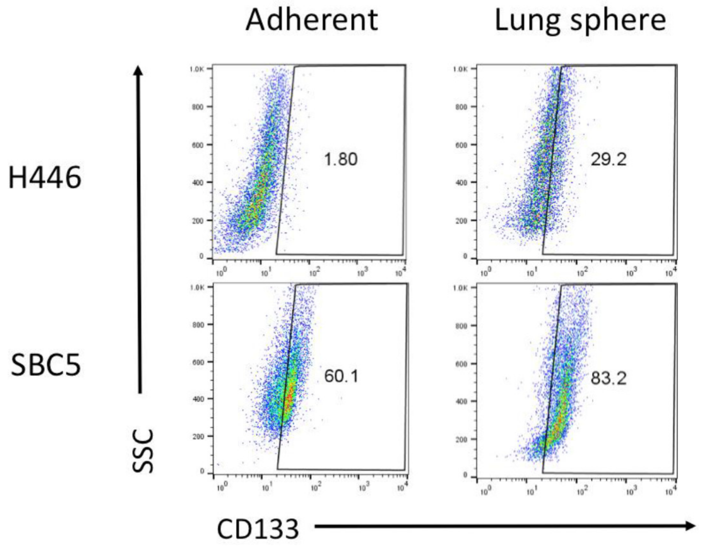

Supplementary Figure S8: CD133 expression is increased in lung sphere (LC) derived from SCLC cells. Adherent H446 and SBC5 SCLC cells were seeded onto the ultra-low attachment 96-well plate and cultured for 8 days. After confirming LS formation, LS mass were dissociated into single cells by gentle pipetting, and they were subjected to flow cytometry analysis to measure CD133 expression levels, in comparison with corresponding parental cells cultured in conventional culture plate.

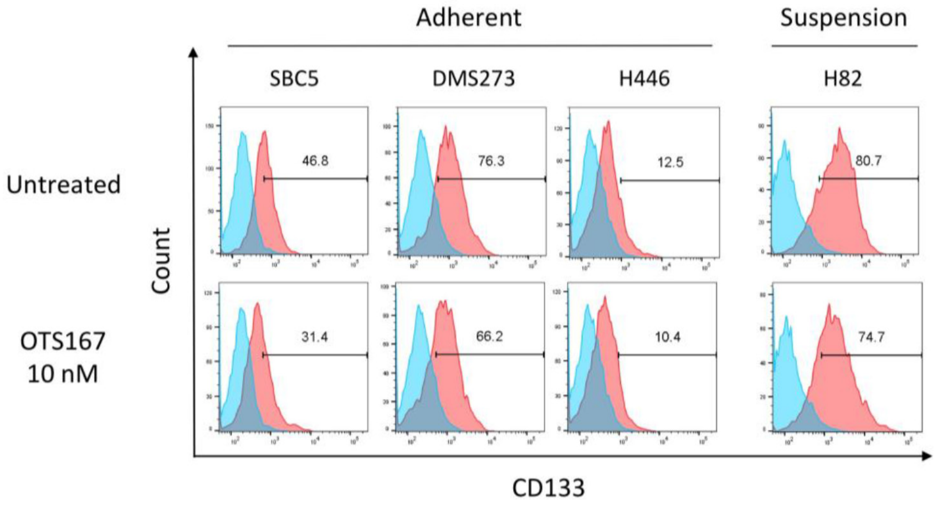

Supplementary Figure S9: Decrease of CD133-positive SCLC cell population by OTS167 treatment. Adherent (SBC5, DMS273 and H446) and suspension (H82) SCLC cells were treated with 10 nM of OTS167 for 48 hours, and then subjected to flow cytometry analysis to measure CD133-positive cell fraction, in comparison with untreated cells.

Supplementary Table S1: Cell authentication of SCLC cell lines by DNA profile

**DNA Marker**	**SBC-3 (JCRB# JCRB 0818)**	**SBC-5 (JCRB# JCRB 0819)**	**DMS 114 (ATCC# CRL-2066)**	**NCI-H446 (ATCC# HTB-171)**	**NCI-H82 (ATCC# HTB-175)**	**NCI-H524 (ATCC# CRL-5831)**
AMEL	X, Y	X, Y	X	X	X	X
CSF1PO	10, 12	10	10, 11	13, 14	11	12
D13S317	11, 12	8, 10	13	8	8	12
D16S539	9	12	12	12	12	12
D5S818	12, 13	10, 11	12	11	12	12
D7S820	12, 13	8, 11	10, 11	10, 11	10, 13	11, 12
TH01	6, 7	6	8, 9.3	8, 9.3	9, 9.3	8, 9.3
TPOX	8, 11	9, 12	8, 11	9, 11	11	8, 10
vWA	18	14, 18	16, 17	18, 19	14	14, 17

ATCC, American Type Culture Collection; JCRB, Japanese Collection of Research Bioresources.
